# The Uses of Advertisement

**Published:** 1920-12-11

**Authors:** 


### THE USES OF ADVERTISEMENT.

ONLY one voluntary hospital possessed a stall at the advertising exhibition; and though a certain amount of loose talk is always current concerning the value of advertisement to hospitals, we doubt if there is any general agreement on the subject, or if the rule of doing what was done last time is not the principle really prevailing at present. There are always two forms of advertisement, the uncommercial and the commercial, and the advertisement of hospitals must remain chiefly in the former class. Its patients, its staff, those whom it trains as students or probationers must be its principle source of reputation. If they do not speak well of it, no advertisement will be of much use. Beside this, there is a natural feeling that institutions supported by charitable funds must not spend more than a limited sum upon advertisement; and thus they are forbidden to enter upon the gigantic gambling in which certain commercial undertakings risk everything for the chance of a big success. It is credibly stated that advertisement can make the success of almost any play; and certainly the popularity of one patent medicine over another of the same pretensions seems to be merely the difference between the extent of the judicious advertisement employed.

This suggests the question whether the practice of advertising for money is conditioned by the same laws which govern the advertisement of goods for sale. We imagine that there is a difference, and that charity advertisements have to be more carefully directed than commercial ones. For example, several hospitals advertise in parish magazines, because these circulate among the charitable, and who else, except local tradesmen, find it worth while to advertise in parish magazines? Except where a hospital offers some service like that of its private nursing staff, it probably gains more from publicity than from advertisement. Publicity chronicles its doings and seeks to interest the public in them. Advertisement states its needs which, to the general public, are less interesting. Consequently publicity has lately been more developed than advertisement; though, in order to induce the lay press to publish hospital news and letters a certain amount of regular advertisement is justified in the lay newspapers. In view, however, of the very large sums of money now required for building, extensions, and to meet overdrafts at the bank, universities and hospitals are considering the question of advertising campaigns on a large scale.

The secretary of the Western Ophthalmic Hospital, Marylebone, which possessed a stall at the advertising exhibition, is reported to be "contracting for advertisement space by which we hope to obtain £50,000 for the rebuilding and modernising of the hospital." Since Mr. H. W. Burleigh also attributed the healthy condition of his hospital's finances to previous advertising, his experiences of the plan would be interesting. In the main we think publicity preferable to advertisement, except that it is necessary for institutions to keep their names and needs before the testators and others who consult charitable organs of opinion before making bequests or gifts. But the fact remains that the value of advertisement to hospitals has not been thoroughly examined, which is unfortunate at a time when money is scarce and can only be urgently spent on methods the value of which has been proved by experience.

